# Non-statin lipid-lowering therapy over time in very-high-risk patients: effectiveness of fixed-dose statin/ezetimibe compared to separate pill combination on LDL-C

**DOI:** 10.1007/s00392-020-01740-8

**Published:** 2020-09-19

**Authors:** Julius L. Katzmann, Francesc Sorio-Vilela, Eugen Dornstauder, Uwe Fraas, Timo Smieszek, Sofia Zappacosta, Ulrich Laufs

**Affiliations:** 1 Klinik und Poliklinik für Kardiologie, Universitätsklinikum Leipzig, Liebigstraße 20, 04103 Leipzig, Germany; 2 AMGEN Europe GmbH, Rotkreuz, Switzerland; 3 AMGEN GmbH, Munich, Germany; 4 IQVIA Technology and Services AG, Basel, Switzerland

**Keywords:** LDL cholesterol, Statin, Ezetimibe, Cardiovascular disease, Adherence, Fixed-dose combination

## Abstract

**Background:**

Many patients at very-high atherosclerotic cardiovascular disease risk do not reach guideline-recommended targets for LDL-C. There is a lack of data on real-world use of non-statin lipid-lowering therapies (LLT) and little is known on the effectiveness of fixed-dose combinations (FDC). We therefore studied prescription trends in oral non-statin LLT and their effects on LDL-C.

**Methods:**

A retrospective analysis was conducted of electronic medical records of outpatients at very-high cardiovascular risk treated by general practitioners (GPs) and cardiologists, and prescribed LLT in Germany between 2013 and 2018.

**Results:**

Data from 311,242 patients were analysed. Prescriptions for high-potency statins (atorvastatin and rosuvastatin) increased from 10.4% and 25.8% of patients treated by GPs and cardiologists, respectively, in 2013, to 34.7% and 58.3% in 2018. Prescription for non-statin LLT remained stable throughout the period and low especially for GPs. Ezetimibe was the most prescribed non-statin LLT in 2018 (GPs, 76.1%; cardiologists, 92.8%). Addition of ezetimibe in patients already prescribed a statin reduced LDL-C by an additional 23.8% (32.3 ± 38.4 mg/dL), with a greater reduction with FDC [reduction 28.4% (40.0 ± 39.1 mg/dL)] as compared to separate pills [19.4% (27.5 ± 33.8 mg/dL)]; *p* < 0.0001. However, only a small proportion of patients reached the recommended LDL-C level of < 70 mg/dL (31.5% with FDC and 21.0% with separate pills).

**Conclusions:**

Prescription for high-potency statins increased over time. Non-statin LLT were infrequently prescribed by GPs. The reduction in LDL-C when statin and ezetimibe were prescribed in combination was considerably larger for FDC; however, a large proportion of patients still remained with uncontrolled LDL-C levels.

**Graphic abstract:**

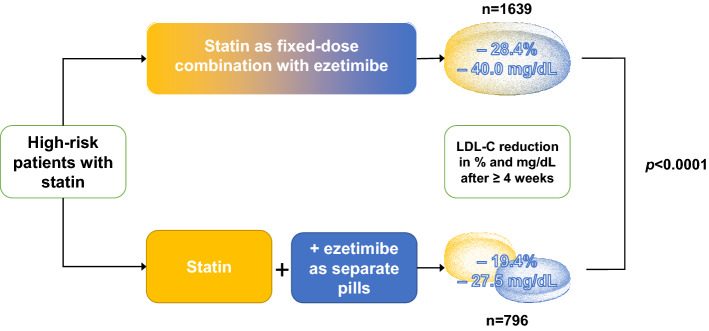

## Introduction

LDL cholesterol (LDL-C) is a modifiable risk factor causally related to atherosclerotic cardiovascular disease (ASCVD) [1, 2]. Strong evidence from clinical trials supports the cardiovascular benefits of lowering LDL-C by statins, cholesterol absorption inhibitors (e.g. ezetimibe), and PCSK9 inhibitors [1]. Major international guidelines therefore recommend lowering of LDL-C to risk-dependent goals to reduce ASCVD risk [3, 4]. However, these treatment targets are only achieved in a minority of patients. Registries such as the EUROASPIRE V survey in 27 European countries report that an LDL-C below 70 mg/dL is achieved in less than a third of very-high-risk patients [5, 6]. Current treatment goals recommended by the European Society of Cardiology (ESC) and the European Atherosclerosis Society (EAS) for these patients are even lower (< 55 mg/dL) [4]. Statins are recommended as first-line therapy to reduce LDL-C. However, in patients with high baseline LDL-C or in patients that cannot tolerate high statin doses, e.g., because of muscle symptoms [7], statin monotherapy may not be sufficient to attain the LDL-C target. This underlines the need for additional non-statin lipid-lowering therapies (LLT) such as ezetimibe [8].

Data on trends in the prescription of oral non-statin LLT may inform real-world utilization of these drugs and help identify opportunities to improve the low rates of LDL-C target attainment. This includes the use of fixed-dose combinations (FDC). In patients with hypertension, FDC have been shown to improve medication adherence and blood pressure control [9, 10]. FDC are therefore recommended by the current ESC guidelines on the treatment of hypertension [11]. However, the available data on FDC for the treatment of dyslipidaemia are sparse.

The main objectives of this study were to analyse (1) prescription trends of oral non-statin LLT in Germany, (2) trends in the prescription of statin/ezetimibe combinations as FDC or separate pill combinations (SPC), and (3) LDL-C reductions achieved with statin/ezetimibe combinations, comparing FDC and SPC in a large cohort of patients at very-high cardiovascular risk.

## Methods

### Data source and study population

This retrospective cohort study was conducted using cross-sectional data obtained from the IMS^®^ Disease Analyzer between January 2013 and December 2018. This database contains anonymised medical records from more than 15 million patients (2019) treated in approximately 2700 outpatient practices equipped with electronic data processing systems in Germany. Data provided by general practitioners (GPs, 936 practices) and cardiologists (62 practices) were analysed, representing 2.2% of all GP and 4.7% of all cardiologist practices. The data available in the IMS^®^ Disease Analyzer are representative of the German population with respect to age, gender, prescription patterns, and chronic diseases such as cancer, dementia, and diabetes [12, 13].

Only data from practices which delivered data for all months throughout a calendar year were included for that respective year. Patients were included if they fulfilled the following criteria:

- older than 18 years of age;
- at least two prescriptions of the same oral lipid-lowering drug within one calendar year (chemical subgroup in the ATC classification system) [14];
- LLT for at least 21 days;
- very-high cardiovascular risk, defined according to the 2016 ESC/EAS guidelines [15] as the presence of at least one of the following comorbidities between 2008 and 2018:
  - cardiovascular disease (defined as at least one diagnosis of angina pectoris, myocardial infarction, chronic heart disease, transient ischaemic attack, stroke, or peripheral arterial disease);
  - diabetes mellitus, type I or II;
  - chronic kidney disease stage IV or V based on ICD-10 codes N18.4 and N18.5, respectively.

Patients were included if prescription events provided complete information at the chemical substance level in the ATC classification system [14]. Combination therapies were identified if the patient received up to two drugs for an overlapping period of at least 21 days. In longitudinal analyses investigating the effect of ezetimibe on LDL-C level, only measurements recorded at least 4 weeks after the first prescription of ezetimibe were included. Antithrombotic use was reported if the patient received at least two prescriptions of antithrombotic agents or anticoagulants of the same class during a calendar year.

As not every practice recorded all patient characteristics, the results only represent the proportion of patients within those with data available. Specifically, information for age and sex were available for most patients, BMI and blood pressure data were available in 20–30% of patients, and lipid profiles were available for 40–50% of patients treated by GPs and 25% of patients treated by cardiologists.

### Statistical analyses

Patient characteristics were summarized in descriptive statistics and are presented stratified by the specialty of the treating physician (GPs or cardiologists) and LLT prescriptions in 2018. Data are presented as mean ± standard deviation. To compare the change in LDL-C after initiation of ezetimibe across different treatment groups, analysis of variance (ANCOVA) was used. A two-sided *p* value < 0.05 was considered statistically significant. Analysis were performed using R (version 3.5.1; R Foundation for Statistical Computing), and SAS (version 9.4; Analytics Software & Solutions).

## Results

### Patient characteristics

**Fig. 1:**
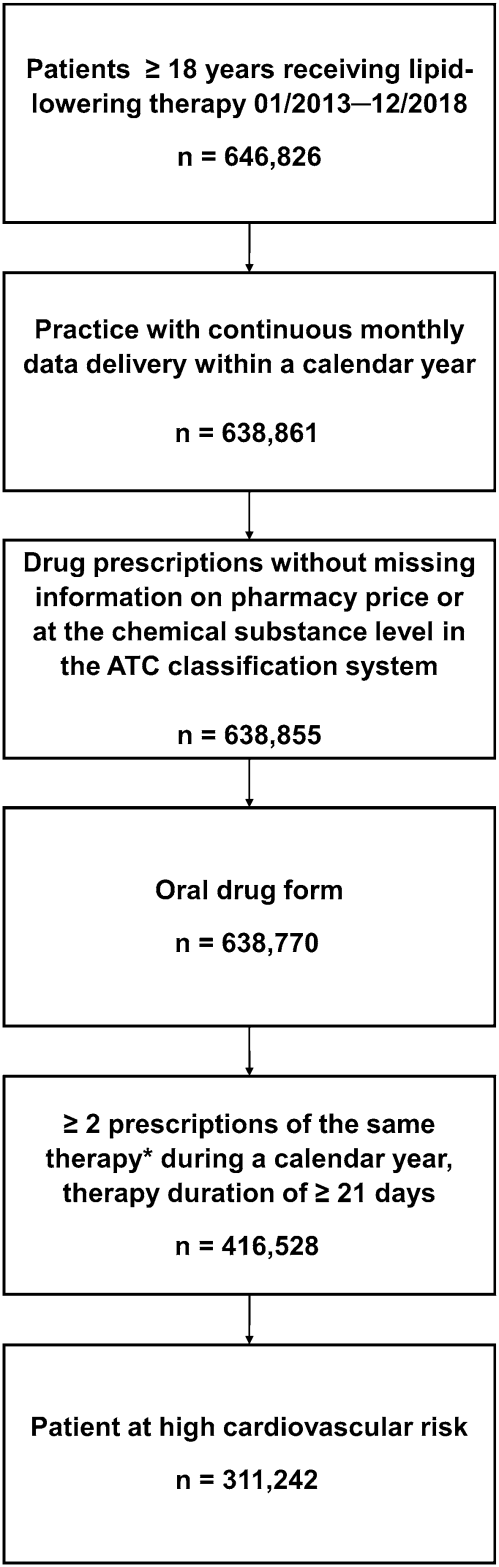
Flowchart of patient selection. * at the chemical subgroup in the ATC classification system

From the total population of 646,826 patients, 311,242 fulfilled the inclusion criteria and were included in the final analyses (Fig. 1). Of the patients prescribed LLT in 2018, 97.2% were treated by GPs and 2.8% by cardiologists. Patient characteristics are presented in Table 1 (GPs) and Table 2 (cardiologists). Mean age was 71.4 and 69.1 years for patients treated by GPs and cardiologists, respectively, 58.8% and 74.6% were male.

**Table 1:** Baseline characteristics for patients treated by general practitioners in 2018

	All lipid-lowering therapies	Statins only	Ezetimibe only	Statin + ezetimibe (separate pills)	Statin + ezetimibe (fixed-dose combination)	Fibrates	Bile acid sequestrants
*n* (%)	136,494 (100)	125,476 (91.9)	1807 (1.3)	533 (0.4)	6429 (4.7)	2093 (1.5)	156 (0.1)
Age	71.4 ± 11.1	71.7 ± 11.1	69.3 ± 10.8	66.1 ± 10.8	67.8 ± 10.5	67.3 ± 12.0	70.9 ± 10.5
Male	80,255 (58.8)	72,895 (58.1)	1106 (61.2)	406 (76.2)	4466 (69.5)	1316 (62.9)	66 (42.3)
BMI	29.6 ± 5.5	29.6 ± 5.5	29.1 ± 5.1	29.2 ± 5.7	29.6 ± 5.2	30.5 ± 5.8	29.8 ± 6.6
Systolic blood pressure	135.8 ± 17.8	135.9 ± 17.9	134.4 ± 16.4	133.4 ± 16.8	134.3 ± 17	138.2 ± 18.2	133.8 ± 18.4
Diastolic blood pressure	78.5 ± 10.1	78.5 ± 10.1	78.9 ± 9.2	78.7 ± 10.2	78.4 ± 9.7	80.4 ± 9.8	79.0 ± 10.5
Risk factors and comorbidities							
ASCVD	98,986 (72.5)	90,910 (72.5)	1482 (82.0)	471 (88.4)	5184 (80.6)	845 (40.4)	94 (60.3)
ACS	15,274 (11.2)	13,613 (10.8)	329 (18.2)	127 (23.8)	1124 (17.5)	74 (3.5)	7 (4.5)
Stroke	20,578 (15.1)	19,655 (15.7)	189 (10.5)	50 (9.4)	547 (8.5)	119 (5.7)	18 (11.5)
TIA	8207 (6.0)	7715 (6.1)	113 (6.3)	21 (3.9)	292 (4.5)	58 (2.8)	8 (5.1)
Aortic aneurysm	188 (0.1)	172 (0.1)	2 (0.1)	0 (0)	11 (0.2)	3 (0.1)	0 (0)
Hypercholesterolaemia	95,161 (69.7)	87,092 (69.4)	1368 (75.7)	413 (77.5)	4744 (73.8)	1469 (70.2)	75 (48.1)
Hypertension	111,507 (81.7)	102,715 (81.9)	1455 (80.5)	411 (77.1)	5126 (79.7)	1678 (80.2)	122 (78.2)
Diabetes mellitus	74,058 (54.3)	68,008 (54.2)	862 (47.7)	201 (37.7)	3362 (52.3)	1554 (74.2)	71 (45.5)
Lipid profile							
Total cholesterol in mg/dL	166.8 ± 38.9	166.7 ± 37.9	183.5 ± 49.8	149.6 ± 35.8	154.9 ± 40.5	199.3 ± 51.4	177.1 ± 40.7
LDL-C in mg/dL	97.6 ± 31.7	97.7 ± 31.0	112.5 ± 41	86.4 ± 29.5	86.4 ± 33.9	122.4 ± 36.4	109.3 ± 34.1
HDL-C in mg/dL	35.1 ± 4.2	35.2 ± 4.2	35.3 ± 4.6	35.3 ± 4.8	34.7 ± 4.4	33.2 ± 5.8	34.9 ± 3.8
Triglycerides in mg/dL	157.2 ± 102.0	154.6 ± 97.2	168.3 ± 126.6	151.3 ± 99.7	172.9 ± 130.5	230.6 ± 166.9	175.1 ± 104.8

**Table 2:** Baseline characteristics for patients treated by cardiologists in 2018

	All lipid-lowering therapies	Statins only	Ezetimibe only	Statin + ezetimibe (separate pills)	Statin + ezetimibe (fixed-dose combination)	Fibrates
*n* (%)	3930 (100)	2999 (76.3)	180 (4.6)	51 (1.3)	682 (17.4)	17 (0.4)
Age	69.1 ± 10.4	69.5 ± 10.4	69 ± 10.1	62.9 ± 9.2	67.9 ± 10.3	65.2 ± 13.6
Male	2930 (74.6)	2199 (73.3)	131 (72.8)	46 (90.2)	540 (79.3)	13 (76.5)
BMI	29.0 ± 5.1	29.2 ± 5.2	27.4 ± 4.5	29.9 ± 3.8	28.4 ± 4.3	31.2 ± 5.2
Systolic blood pressure	136.0 ± 18.3	136.7 ± 17.6	136.4 ± 30.1	134.8 ± 19.5	129.4 ± 16.2	142.4 ± 15.9
Diastolic blood pressure	78.9 ± 9.0	79.0 ± 9.0	79.4 ± 9.3	79.4 ± 8.5	77.4 ± 8.3	81 ± 8.2
Risk factors and comorbidities						
ASCVD	3409 (86.7)	2546 (84.9)	170 (94.4)	49 (96.1)	636 (93.3)	7 (41.2)
ACS	395 (10.1)	275 (9.2)	20 (11.1)	6 (11.8)	93 (13.6)	1 (5.9)
Stroke	201 (5.1)	160 (5.3)	9 (5.0)	0 (0)	31 (4.5)	1 (5.9)
TIA	88 (2.2)	71 (2.4)	8 (4.4)	1 (2.0)	8 (1.2)	0 (0)
Aortic aneurysm	3 (0.1)	3 (0.1)	0 (0)	0 (0)	0 (0)	0 (0)
Hypercholesterolaemia	2737 (69.6)	2040 (68)	130 (72.2)	34 (66.7)	518 (76)	14 (82.4)
Hypertension	2935 (74.7)	2265 (75.5)	125 (69.4)	38 (74.5)	495 (72.6)	11 (64.7)
Diabetes mellitus	1165 (29.6)	941 (31.4)	44 (24.4)	9 (17.6)	159 (23.3)	12 (70.6)
Lipid profile						
Total cholesterol in mg/dL	160.6 ± 37.1	160.1 ± 35.6	177.8 ± 42.7	168.4 ± 37.4	153.7 ± 41.6	181.9 ± 44.7
LDL-C in mg/dL	95.5 ± 29.5	95.7 ± 28.4	109.8 ± 34.9	86.8 ± 32.0	85.4 ± 30.7	120.1 ± 30.1
HDL-C in mg/dL	35.1 ± 4.1	35.1 ± 4.3	35.3 ± 3.6	34 ± NA	34.9 ± 3.3	37.3 ± 3.8
Triglycerides in mg/dL	146.7 ± 88.6	144.6 ± 85.7	150.3 ± 82.7	146 ± 61.7	160.1 ± 115.8	187.8 ± 95.7

None of the patients treated by cardiologists received bile acid sequestrants

Plus-minus values are mean ± SD, other values are *n* (%), or as indicated

*BMI* body-mass index, *ASCVD* atherosclerotic cardiovascular disease, *ACS* acute coronary syndrome, *TIA* transient ischaemic attack, *NA* not available

The most frequent cardiovascular risk factors in patients treated by GPs and cardiologists, respectively, were hypertension (81.7% and 74.7%), diagnosed hypercholesterolaemia based on the ICD-10 code (69.7% and 69.6%), and type 2 diabetes (54.3% and 29.6%). 11.2% and 10.1% had a history of an acute coronary syndrome, and 21.1% and 7.4% had a history of stroke or transient ischaemic attack. The proportion of patients with ASCVD was higher in patients treated by cardiologists than in those treated by GPs (86.7% vs. 72.5%).

Mean systolic and diastolic blood pressure was 135.8 and 78.5 mmHg in patients treated by GPs, and 136.0 and 78.9 mmHg in patients treated by cardiologists. Mean LDL-C was 97.6 and 95.5 mg/dL in patients treated by GPs and cardiologists, respectively.

In general, baseline characteristics were similar across patients prescribed the different categories of LLT. However, compared with patients prescribed statin monotherapy, the prevalence of ASCVD was higher in patients prescribed ezetimibe (monotherapy or in combination with statins as FDC or in separate pills) and lower in patients prescribed fibrates. This trend was observed among patients treated by GPs and cardiologists.

### Prescription trends in lipid-lowering therapies

**Fig. 2:**
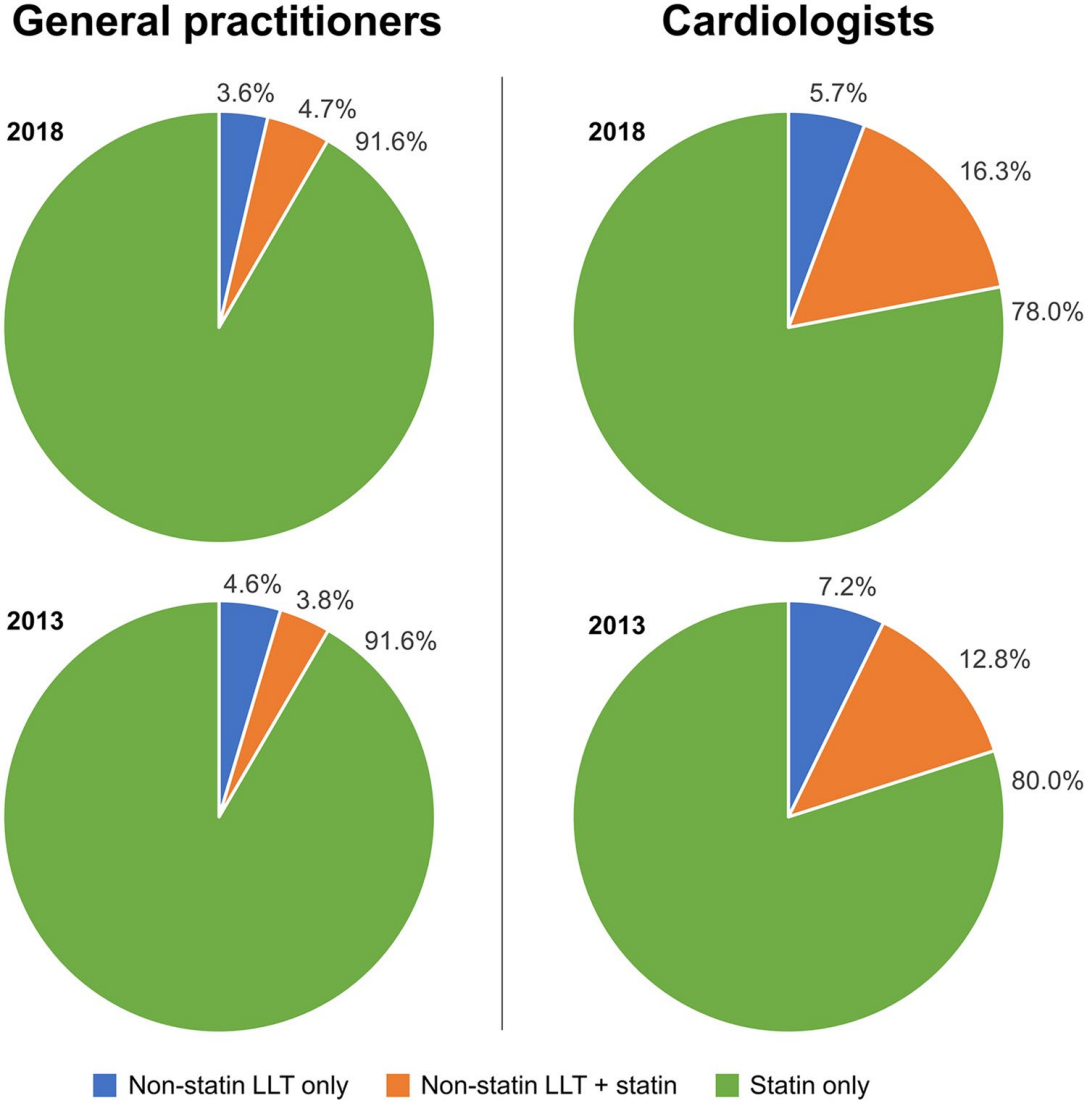
Prescription trends in lipid-lowering therapy 2013–2018. *LLT* lipid-lowering therapy

Prescription rates for LLT in 2013 and 2018 are summarized in Fig. 2.

Prescriptions for statin monotherapy were higher among patients treated by GPs (91.6%, based on average from the years 2013 to 2018) compared with patients treated by cardiologists (79%), with no significant change from 2013 to 2018. Prescription rates for non-statin LLTs were also stable during the study period and higher in patients treated by cardiologists. In patients treated by cardiologists, prescription rates for statin/non-statin combination LLT increased from 12.8% in 2013 to 16.3% in 2018. This increase was partially balanced by a decrease from 7.2% to 5.7% (2013/2018) in the use of non-statin LLT monotherapy.

#### Statins

**Fig. 3:**
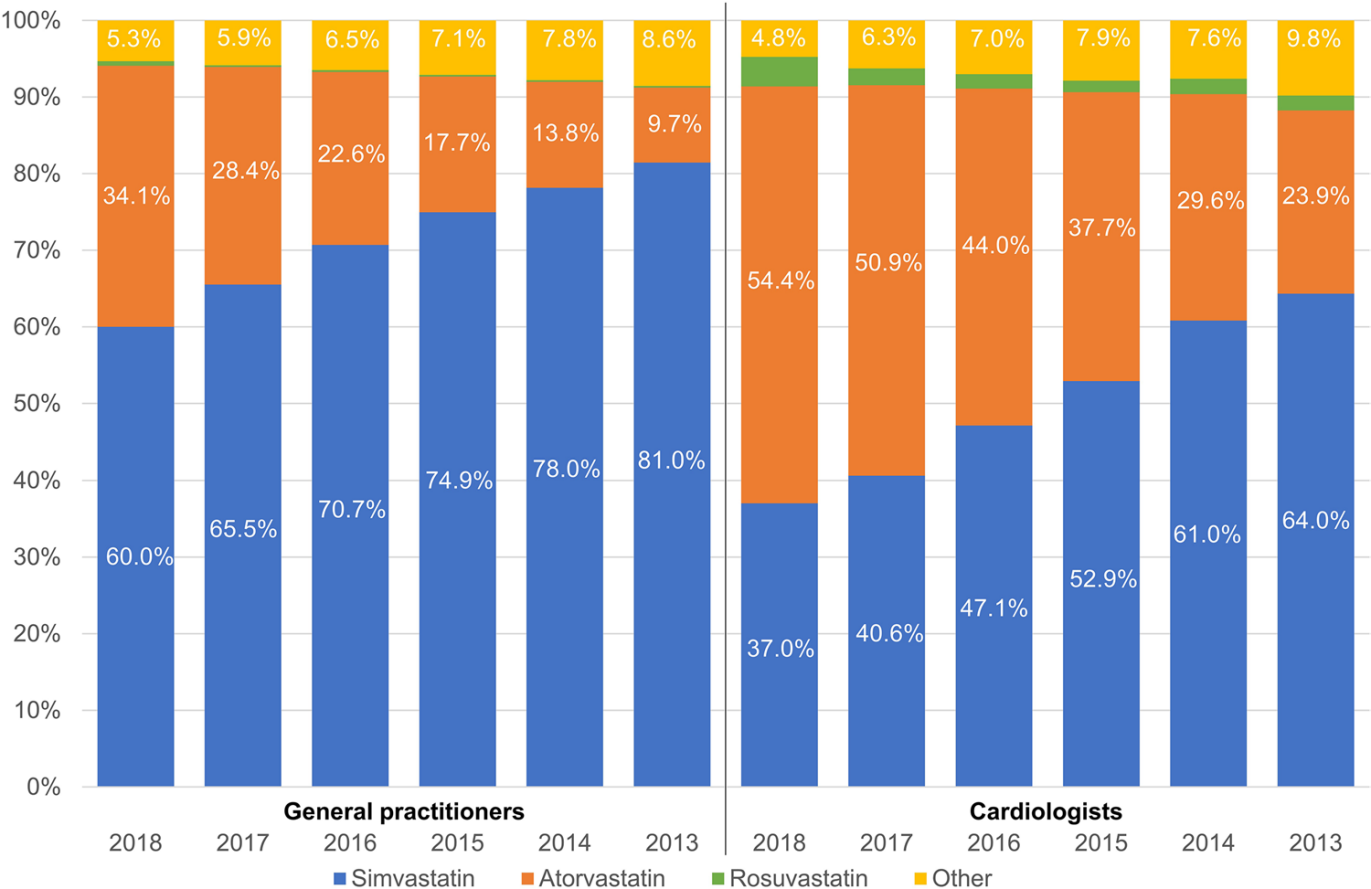
Prescription trends in statins 2013–2018

Trends in statin prescriptions are shown in Fig. 3.

During the observation period, prescriptions of high-potency statins (atorvastatin and rosuvastatin) increased, while prescriptions of simvastatin decreased. Prescription rates for other statins were low. In agreement with previous analyses [16], the majority of statins were used in low and moderate doses. While overall trends were comparable, prescription rates of high-potency statins were higher among patients treated by cardiologists compared to GPs (58.3% and 34.7% of patients respectively). There was no significant change of the prescribing pattern of statin doses by GPs between the year 2013 and 2018 with the exception of an increase of high-dose atorvastatin from 35.9 to 43.3%. In cardiology practice, the use of high doses of atorvastatin and of rosuvastatin increased from 40.8 to 50.2% and 31.6 to 43.1% between 2013 and 2018, respectively.

#### Non-statin lipid-lowering therapies

**Fig. 4:**
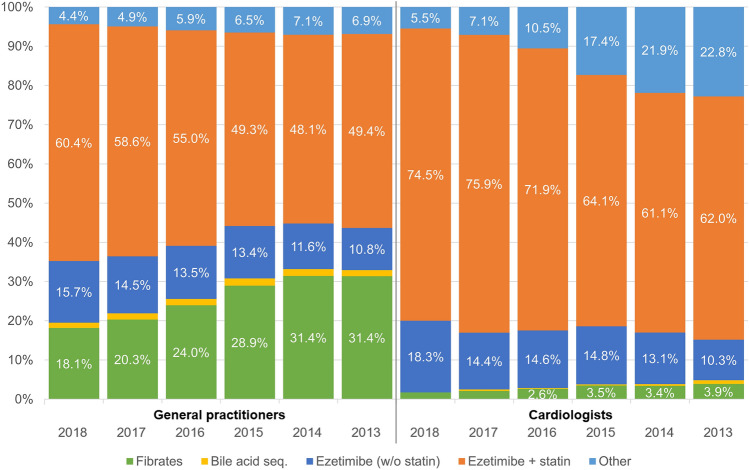
Prescription trends in non-statin lipid-lowering therapies 2013–2018. *sequ*. sequestrants, *w/o* without

Trends in prescriptions for non-statin LLT are shown in Fig. 4.

Prescriptions of ezetimibe increased steadily in the period from 2013 to 2018; in the majority of cases, it was prescribed in combination with statins. In 2018, 60.4%/74.5% of all non-statin LLT prescriptions were statin/ezetimibe combinations, and 15.7%/18.3% ezetimibe monotherapy, as prescribed by GPs and cardiologists, respectively.

The increase in ezetimibe prescriptions by GPs and cardiologists was balanced by a decrease in prescriptions for fibrates among GPs and a decrease in prescriptions for non-statin LLT other than ezetimibe, fibrates or bile acid sequestrants among cardiologists.

The patterns of LLT prescriptions by GPs were comparable between the areas of former West and East Germany.

### Prescription trends of ezetimibe on top of statin therapy

**Fig. 5:**
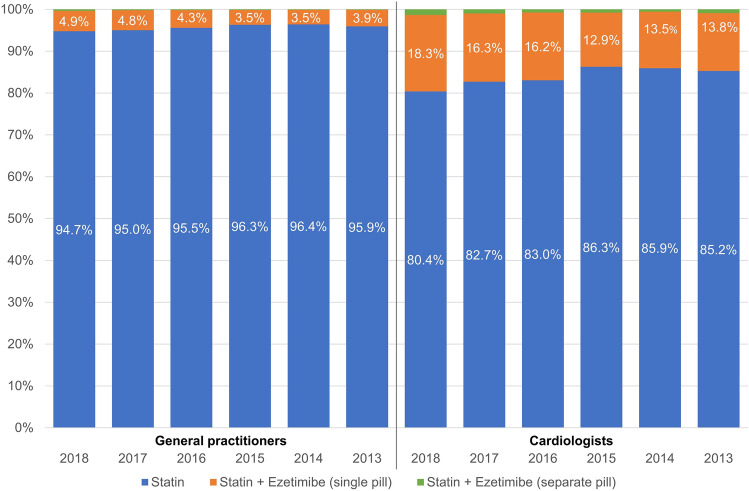
Prescription trends in statins and statin–ezetimibe combinations

Ezetimibe was the most frequently prescribed non-statin LLT, with an increase in prescriptions in the period analysed (Fig. 4) both for GPs and cardiologists. Figure 5 shows the proportion of all patients with a prescription for statin monotherapy and statin/ezetimibe combination therapy, with stratification by formulation as FDC or separate pills (SPC). From 2013 to 2018, ezetimibe prescriptions on top of statin therapy increased in patients treated by cardiologists, with 19.6% of patients prescribed statins also prescribed ezetimibe in 2018. In contrast, this proportion was lower in patients treated by GPs with a negligible increase in ezetimibe prescriptions on top of statin therapy (5.3% of the patients receiving ezetimibe on top of statin therapy in 2018). The majority of patients prescribed a statin/ezetimibe combination received an FDC (GPs: 92.3%, cardiologists: 93.0%), and only a minority received SPC (GPs: 7.7%, cardiologists: 7.0%). This pattern was comparable in patients treated by GPs and cardiologists.

### Effect of statin/ezetimibe combinations as single (FDC) or separate pills (SPC) on LDL-C

**Fig. 6:**
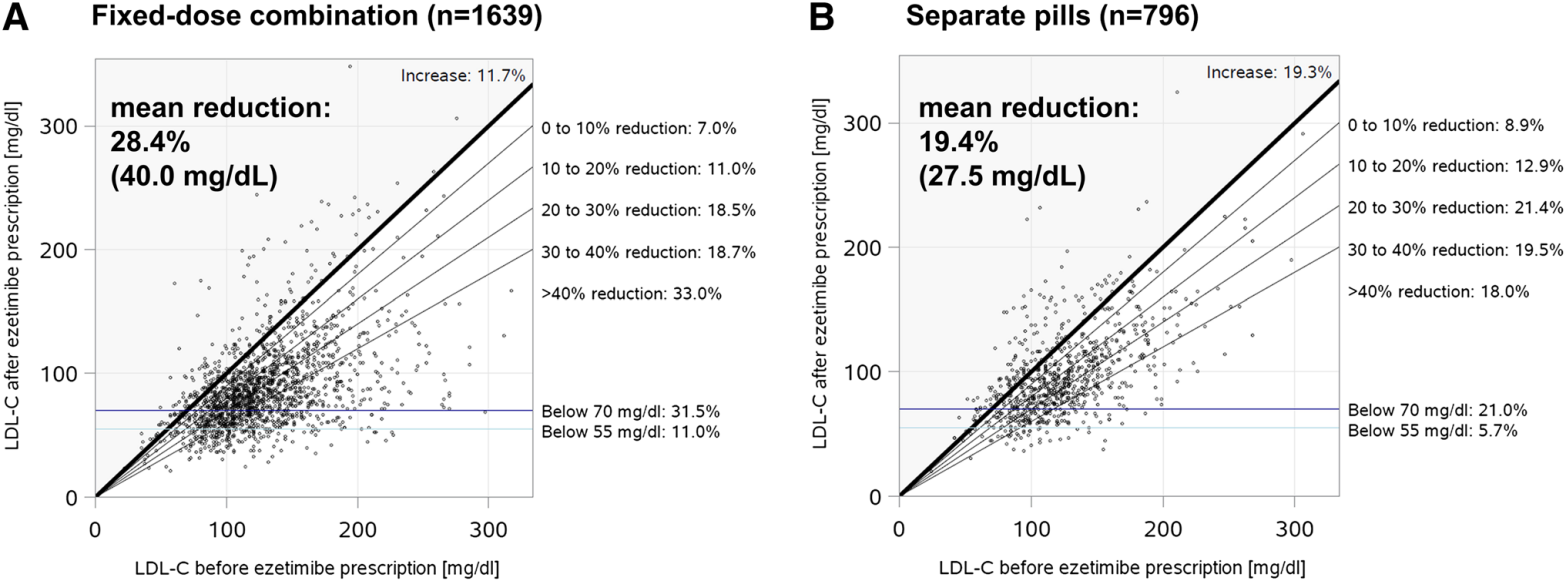
LDL-C reduction after initiation of ezetimibe as fixed-dose combination (FDC; **a**) or separate pills (SPC; **b**). Comparison of LDL-C reduction after ezetimibe initiation for FDC vs. SPC: *p* < 0.0001. Diagonal lines show the percentage of patients that achieved an LDL-C reduction within a specific range. Horizontal lines show the percentage of patients achieving LDL-C control defined as < 70 and < 55 mg/dL, respectively. *LDL-C* low-density lipoprotein cholesterol

Mean LDL-C before and after ezetimibe prescription is shown in Fig. 6. Overall, mean LDL-C reduction following the addition of ezetimibe prescription to statins was 23.8% (32.3 ± 38.4 mg/dL). Among patients prescribed a statin and ezetimibe as FDC, mean LDL-C reduction was 28.4% (40.0 ± 39.1 mg/dL). In contrast, LDL-C was reduced by 19.4% (27.5 ± 33.8 mg/dL) in patients prescribed statin and ezetimibe as separate pills. This difference was highly significant (*p* < 0.0001; Graphic abstract).

The larger reduction of LDL-C resulted in better LDL-C control, although a small proportion of patients reached the recommended LDL-C levels. Of the patients with FDC treatment, 31.5% reached an LDL-C level < 70 mg/dL (recommended by ESC at that time [15, 17]), as compared to 21.0% of the patients receiving their medication as separate pills. The proportion of patients reaching recommended LDL-C target levels would have been even lower (11.0% for the FDC and 5.7% for the separate pills combinations) using the more recent ESC-recommended LDL-C level of < 55 mg/dL for very-high-risk patients [4].

## Discussion

In this analysis of 311,242 German outpatients at very-high risk of ASCVD, prescriptions for high-potency statins increased from 2013 to 2018. Prescriptions for non-statin LLT remained stable over the study period, with an increase in ezetimibe prescriptions balanced by a decrease in fibrates prescriptions among GPs and a decrease in prescriptions for other non-statin LLTs among cardiologists. The most frequently prescribed and increasingly utilized non-statin LLT was ezetimibe. An important and novel finding is that LDL-C reduction was significantly greater when statin and ezetimibe were prescribed as fixed dose combination (FDC) compared with separate pill combinations (SPC). Our study shows the potential of lipid-lowering combination therapy and suggests that, similar to current recommendations for anti-hypertensive treatment, FDC prescription should be preferred over SPC.

The current ESC/EAS guidelines for the management of dyslipidaemias [4] introduced an LDL-C target of 55 mg/dL for very-high-risk patients. While statins remain the cornerstone of lipid lowering in patients at very-high cardiovascular risk, current LDL-C treatment targets will not be reached in many patients receiving statin monotherapy, and additional non-statin LLT is needed [18]. Our study confirms the effectiveness of ezetimibe in lowering LDL-C in a real-world setting. The observed relative LDL-C reductions are comparable to those seen in the IMPROVE-IT trial [19]. Our data therefore support the use of combination LLT as an important strategy to improve LDL-C target attainment in the ASCVD population.

Another important and novel finding is the larger proportional and absolute LDL-C reduction observed in patients receiving statin/ezetimibe FDC as compared to SPC, resulting in a higher percentage of patients achieving LDL-C target. In hypertension, a reduction in the number of tablets prescribed is associated with better medication adherence and blood pressure control [10]. An analysis from an Australian health dataset found no association between statin/ezetimibe FDC and medication possession ratio; however, this study did not assess cholesterol effects [20]. In contrast, in the treatment of hypertension, there are several studies showing improved medication adherence under FDC treatment compared to SPC [9, 10]. Moreover, higher adherence is associated with lower LDL-C levels [21, 22], and adherence to lipid-lowering and blood pressure-lowering therapies is strongly correlated [22]. While our study cannot prove the underlying cause of improved LDL-C lowering with FDC, the aforementioned data suggest better medication adherence is a likely explanation. However, it is relevant to note that, following the addition of ezetimibe to statins and despite the additional decrease in LDL-C, few patients with very-high cardiovascular risk achieved the recommended LDL-C levels. Our data suggest that, in addition to ezetimibe, many patients with very-high cardiovascular risk will require the addition of high-potency therapies such as PCSK9 inhibitors to bring LDL-C levels below the recommended targets.

Our results highlight the importance of medication adherence for chronic conditions, which has been associated with mediators of ASCVD such as LDL-C or blood pressure, and also with mortality [23–25]. Considering the same amount of drug is considerably more efficacious when provided as FDC, our findings highlight the importance of strategies to support medication adherence. In this respect, it appears beneficial to introduce novel non-statin oral LLT as FDC formulations [26]. Identification of patients at risk of low adherence, such as patients with depression, may inform prescription choices [22] and measures to improve medication adherence should gain more attention [27].

Our study has limitations. We do not have data on medication adherence which would strengthen our conclusion. Furthermore, the reasons why physicians prescribed FDC or SPC cannot be derived from the data, potential certain confounding related to the prescribing individuals is possible and could only be excluded by a randomized trial. Strengths of our study are the inclusion of a very large and representative cohort and the long observation period. Another strength of the dataset is the longitudinal follow-up of LDL-C that allows for inferences on changes in parameters due to changes in medication.

In conclusion, we provide definitive information regarding trends in LLT prescriptions in Germany from 2013 to 2018. Prescriptions for high-intensity statins and ezetimibe increased over time. Ezetimibe added to statin therapy effectively reduced LDL-C and increased the proportion of patients with controlled LDL-C, with FDC treatment being more effective than SPC. These data identify practical strategies to improve LDL-C goal achievement for ASCVD prevention. However, a high proportion of patients remain with uncontrolled LDL-C despite the combination of oral LLT.

## Data Availability

Data are available upon request by IQVIA.
